# Bibliographical

**Published:** 1860-11

**Authors:** 


### Bibliographical.

"*THE INSTITUTE OF MEDICINE:*" By MARTYN PAINE, A. M., M. D., L. L. D., Prof. of the Institutes of Medicine and Materia Medica in the University of New York. Publishers, Harper & Bro.'s, Franklin Square, New York.

We have received the fifth edition of this work, which has rapidly followed the fourth, the latter, we are informed having been entirely exhausted. Having upon a former occasion given our views in reference to this work, we do not propose now to go into any lengthy review, but merely to reiterate the favorable opinion formerly expressed, and to give our testimony to the correctness of what we believe to be the almost unanimous voice of the profession, that it is an exceedingly able and elaborate work, from one of the most learned medical men of the United States. While we do not agree with the author in all his views, we have no sympathy with the falsification and perversions of his statements and opinions which have appeared in some of the journals under the name of "Reviews." In the present edition we find some remaining typographical errors corrected, numerous references to sections introduced, and a *supplement* added. It is one of those medical works which should by all means be in the possession of every medical man, and as such we commend it. Whilst it may not be very creditable to some of the recipients, we would remark in conclusion, that it has furnished the materials for a large amount of reputation to others beside its author, to which he would doubtless have had no objection, if the source of information had been acknowledged.

We have also received from Messrs. Blanchard & Lea "A Practical Treatise on the Diseases of the Lungs, including the Principles of Physical Diagnosis, by Walter Hoyle Walshe, M. D., London." Being a new *American*, from the third revised and much enlarged English edition of a work of established reputation. The theory of various accoustic phenomena has been examined afresh; and an attempt made to establish the practice of *Percussion* on a new and it is hoped, true and clinically useful system than that hitherto adopted. Descriptions of several diseases, previously omitted, are now introduced; the causes and mode of production of the more important affections so far as they possess directly practical significance are succinctly inquired into; an effort has been made to bring the description of anatomical characters to the level of the wants of the student, as well as of the practical physician, and the diagnosis and prognosis of each complaint are more completely considered. We find also that the sections on Treatment and the Appendix (concerning the influence of climate on pulmonary disorders,) have been partly enlarged, and altogether, the work is such a one as will prove of signal advantage to those who are really willing to study this important class of disease.

Messrs. Blanchard & Lea, of Philadelphia, also send "Ashton on Diseases of the Rectum and Anus," containing an account of Injuries and Malformations of those parts, with remarks on Habitual Constipation. The work embraces chapters on Irritation and Itching of the Anus—Inflammation and Excoriation of the Anus—Excrescences of the Anal Region—Contraction of the Anus—Fissure of the Anus and lower part of the Rectum—Neuralgia of the Anus and Extremity of the Rectum—Inflammation of the Rectum—Ulceration of the Rectum—Hemorrhoidal Affections—Enlargement of Hemorrhoidal Veins—Prolapsus of the Rectum—Abscess near the Rectum—Fistula in Ano—Polypi of the Rectum—Stricture of the Rectum—Malignant Diseases of the Rectum—Injuries of the Rectum—Foreign Bodies in the Rectum—Malformations of the Rectum, and Habitual Constipation. These chapters embrace descriptions and treatment of a class of Diseases than which, in civilized communities, none are more prevalent, cause more suffering, or induce so many varied and distressing sympathetic affections. For an accurate knowledge and a judicious treatment of these affections we would recommend this book.

We have also from Messrs. Ticknor and Fields, Boston, "Electro-Physiology and Electro-Therapeutics;" showing the best method for the Medical Uses of Electricity, by Alfred C. Garratt, M. D., Fellow of the Massachusetts Medical Society, &c.

A systematic work on the Medical and Surgical Uses of Electricity has been greatly needed. The book before us professing to contain clear and practical directions as to *where*, WHEN and *how* to employ electricity as a *remedy*, and to contain at the same time, the condensed scope of those natural, accidental, as well as artificial electric influences, that affect life and health. Our own knowledge upon this subject is too deficient to enable us to pronounce understandingly whether there has been a faithful performance of the task imposed. It seems however to be a sensible work from a respectable source, and as such we would call the attention of the profession to it.

The last work we notice is received from those enterprising and extensive publishers, Messrs. Blanchard & Lea, Philadelphia, and is entitled, "Obscure Diseases of the Brain, and Disorders of the Mind—their Incipient Symptoms, Pathology, Diagnosis, Treatment, and Prophylaxis, by Forbes Winslow, M. D., &c., &c., London."

This work of nearly 600 pages, is intended to be a mere *resume* of the more prominent incipient symptoms of the various forms of cerebral and mental disorder, and was originally intended as an introductory chapter to a treatise on "Softening and other types of Organic Disease of the Brain," which became gradually developed into a large and distinct volume; but is now only intended as an *avant courier*, or introduction to the work which is to relate exclusively to the Specific and individual types of encephalic disease. This is an exceedingly valuable work, especially in connection with the great importance of recognizing cerebral and mental disorders in their *early* stage, this being the only period at which they can be treated with any prospect of success, as suggested in this work in an extract from Dr. F. Hawkins. "In acute cases the period is brief indeed, in which the power of art is available. But whether the case be acute or chronic, it is only in the early stage that its precise nature admits of being distinguished with accuracy. In its further progress, from the extensive sympathies of the brain with other parts of the body, so many functions become implicated and so various are the symptoms which arise as to preclude arrangement or classification and defy the art of diagnosis. The aid which in most other cases, the sensations of the patient are capable of affording us, is lost to us too soon in discorders of the head, until in their advanced state, they all resemble one another, and present alike a dreary abolition of the powers of animal life. The period is therefore highly precious in which these affections admit of being distinguished with precision or treated with any hope of advantage." Upon which the author remarks: "Let the physician then estimate, in all its vital importance, the grave necessity for prompt treatment and decesive remedial measures, when satisfied that the enemy is at the gates and has attacked or is on the eve of assaulting, the citadel; under these circumstances, hesitation, delay or pro-crastination in bringing the patient within the range of sanative measures is fraught with the direst results, and with the saddest consequences. Let us not wilfully close our eyes to the premonitory signs, however apparently insignificant, slight, transient and fugitive they may appear, of actual mental disorder and brain disease, for it is in this early stage when so much may be affected by judicious medical treatment to obstsuct the advance of the fatal cerebral mischief." Too much importance can not be attached to the only symptoms which indicate disorders of this charcter and, as a most valuable aid in detecting the first manifestations of cerebral disease we would recommend this work of Dr. Winslow.

